# Prescription drug misuse and risk factors among Somali adolescents: a qualitative study exploring peer influence, stress, and academic pressure

**DOI:** 10.1186/s12888-025-07336-8

**Published:** 2025-09-30

**Authors:** Mohamed Jayte, Abdifitah Abdullahi Mohamed, Abdifatah Hersi Karshe, Hassan Omar Ali, Abdullahi Hussein Ahmed

**Affiliations:** 1 Internal Medicine Department, Kampala International University, P.O Box 7062 Kampala, Uganda; 2 Microbiology Department, Kampala International University, Kampala, Uganda

**Keywords:** Drug misuse, Adolescents, Somalia, Peer pressure, Academic stress, Prescription drugs

## Abstract

**Background:**

Drug misuse among adolescents is a growing public health concern in Somalia, with prescription drug abuse emerging as a significant issue. This behavior is influenced by a complex interplay of psychological, social, and environmental factors, including peer pressure, academic stress, and community dynamics. Understanding these factors is essential for designing effective interventions to mitigate the rising trend of substance abuse among Somali youth.

**Methods:**

This qualitative study employed in-depth interviews (IDIs) and key informant interviews (KIIs) to explore the motivations and risk factors associated with drug misuse among Somali adolescents. A purposive sampling technique was used to recruit 20 participants, including 15 adolescents and 5 key informants (parents, teachers, and community leaders). Data were analyzed using NVivo software, with thematic analysis conducted to identify emerging themes.

**Results:**

The study identified multiple risk factors contributing to adolescent drug misuse. Psychological stressors, such as anxiety and academic pressure, were key motivations for drug use. Social influences, particularly peer pressure and digital platforms, played a significant role in drug initiation. Family dynamics, including parental neglect and socioeconomic challenges, further exacerbated the problem.

**Conclusion:**

Addressing adolescent drug misuse in Somalia requires multi-level interventions, including family support, school-based awareness programs, and policy enforcement. Community engagement is crucial in preventing substance abuse among Somali youth.

## Background

Adolescent prescription drug misuse is a significant and growing public health challenge globally. The World Health Organization (WHO) estimates that approximately 13% of adolescents worldwide engage in the non-medical use of prescription medications such as opioids, stimulants, and sedatives [1]. In high-income countries like the United States, surveys have documented misuse rates of 5–8%, with common motivations including academic performance enhancement, stress relief, and recreational use [2]. These behaviors are often reinforced by social media, peer norms, and easy access to pharmaceuticals.

In Africa, the issue is increasingly documented in countries such as Kenya, Ethiopia, and Nigeria. Tramadol misuse has been reported among secondary school students and university youth, often linked to academic pressure and peer influence [3]. In Ethiopia, for example, a study found that 12.9% of university students reported using prescription drugs without a medical need [4]. Yet, many African countries lack adequate surveillance systems and regulatory controls to monitor prescription medication distribution or usage among adolescents, allowing the problem to grow largely unchecked.

In Somalia, the situation is particularly concerning due to the fragile healthcare system, ongoing socio-political instability, and lack of regulatory enforcement. According to a 2023 report by the United Nations Office on Drugs and Crime (UNODC), over 68% of pharmacies in Mogadishu dispense prescription medications—such as tramadol (opioid), diazepam (benzodiazepine), and methylphenidate (stimulant)—without requiring prescriptions [5]. These drugs are used for a variety of reasons: tramadol for relaxation or mood enhancement, diazepam for anxiety, and methylphenidate as a “study drug” among students under exam pressure. Unlike khat (Catha edulis), which is traditionally used in Somali society and better documented, the misuse of prescription drugs remains largely unexplored in formal research [6].

Despite anecdotal and institutional reports suggesting a sharp rise in adolescent misuse of prescription drugs in Somalia, there is a critical lack of empirical evidence to understand the scale and underlying drivers of this behavior. This gap hinders the design of effective interventions tailored to Somali adolescents’ needs.

Somali adolescents are increasingly exposed to prescription drugs without adequate regulatory safeguards, mental health support, or parental supervision [7, 8]. The existing literature focuses primarily on alcohol or khat use, leaving a critical knowledge gap regarding prescription drug misuse. This study seeks to address that gap by exploring the psychological, social, academic, and structural factors contributing to misuse in the Somali urban context.

By employing a qualitative design grounded in Bronfenbrenner’s Ecological Systems Theory, this study aims to provide a nuanced understanding of how adolescents’ behaviors are shaped by multi-level influences, including peers, families, schools, and socio-economic environments [9–11]. The findings are expected to guide future interventions, policy frameworks, and community-based strategies targeting youth substance misuse in fragile-state settings like Somalia. The study aimed to explore the risk factors, motivations, and socio-cultural dynamics contributing to prescription drug misuse among Somali adolescents, with a focus on peer influence, academic stress, and community risk environments.

## Methods

Prescription drug misuse is defined as the use of prescription medication in a manner other than prescribed, including using it without a prescription, in higher doses, for longer durations, or for non-medical purposes such as to experience euphoria [12].

### Study setting and period

The study was conducted in Mogadishu, Somalia, between January and April 2024. Mogadishu was selected as the primary study site due to its high prevalence of adolescent prescription drug misuse, as reported by municipal health authorities. The city’s urban density, socioeconomic diversity, and concentration of educational and healthcare institutions provided a representative context for examining risk factors such as peer networks, academic stress, and community dynamics.

### Study design

A descriptive qualitative study design was adopted, adhering to the Consolidated Criteria for Reporting Qualitative Research (COREQ) guidelines. This approach prioritized an in-depth exploration of participants’ lived experiences and contextual challenges. Ethical approval was secured from the Minister of health Review Board (Ref: SNU-IRB-2025-045). Written informed consent was obtained from adult participants, while parental consent and adolescent assent were acquired for minors.

### Participant selection

Purposive sampling was used to recruit 15 adolescents (aged 14–19) with a self-reported history of non-medical prescription drug use (e.g., opioids, stimulants, sedatives) within the past six months. Participants were identified through a multi-stage process. First, three secondary schools in Hodan and Hamar-Weyne districts, areas with high reported drug misuse, were selected, and teachers referred students exhibiting behavioral or academic declines linked to substance use. Second, two public clinics in Wadajir District, known for treating adolescent overdose cases, identified potential participants. Finally, a local youth welfare NGO in Howlwadag District connected researchers with adolescents self-reporting drug misuse. Drug misuse was identified through self-report during screening interviews. Participants were also referred by school teachers and clinic staff based on behavioral concerns or prior overdose history. No formal diagnostic interviews (such as Structured Clinical Interview for DSM- SCID) were used, which is acknowledged as a limitation.

Inclusion criteria: Adolescents aged 14–19, living in Mogadishu, with self-reported non-medical use of prescription drugs (e.g., tramadol, methylphenidate, diazepam) in the past six months, and fluency in Somali Exclusion of adolescents with severe cognitive or psychological conditions was based on teacher and parent reports. Due to field limitations, no formal tools (e.g., MMSE, PHQ-9) were used.

### Data collection

**Fig. 1:**
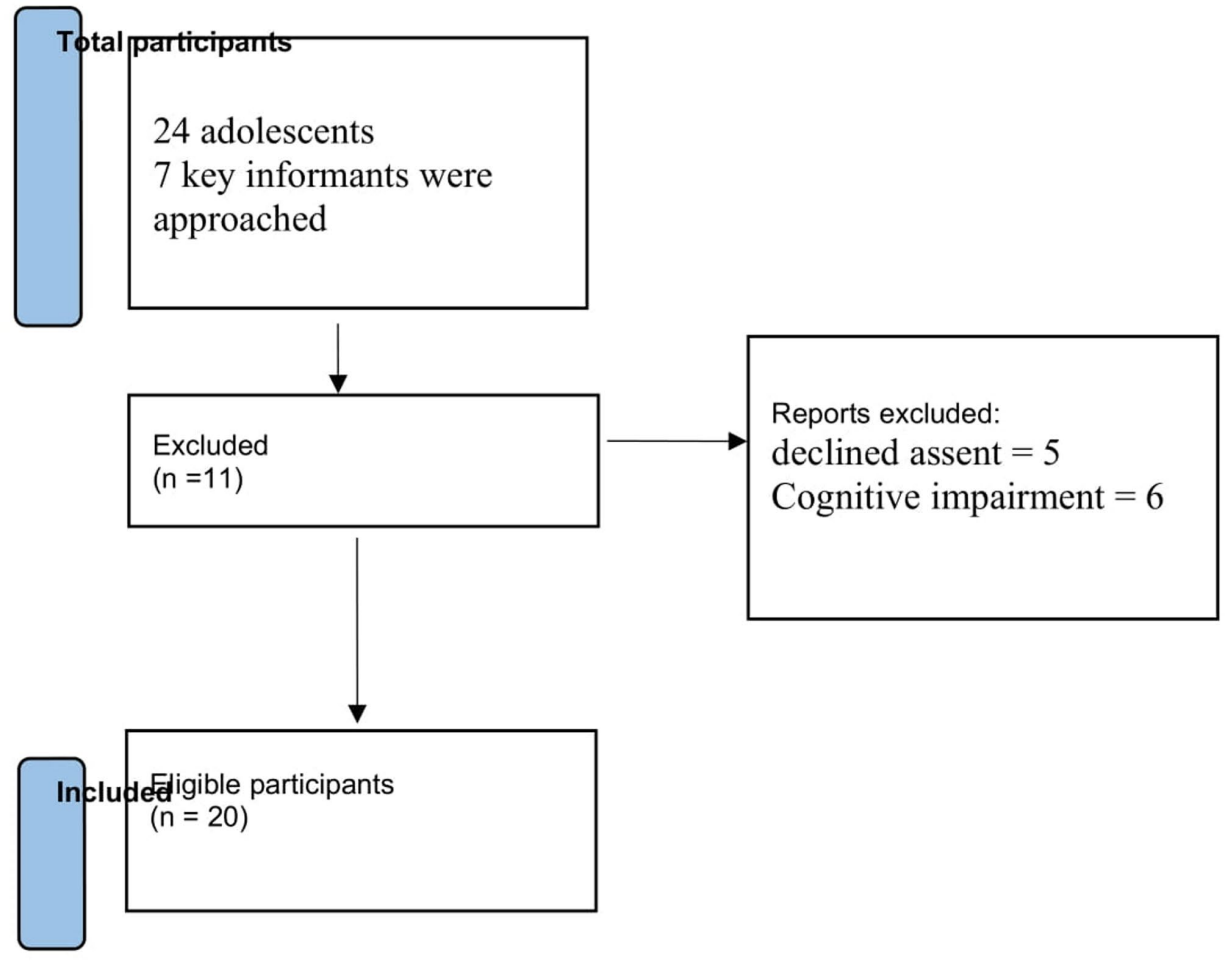
Showing Prisma chart

Figure 1 presents a PRISMA flow diagram adapted for this qualitative study, outlining the screening and selection process for participants. This provides transparency on how initial contacts were screened, excluded, or included. A total of 24 adolescents and 7 key informants were approached. 6 adolescents were excluded due to ineligibility, 3 declined assent, and 2 parents declined consent. 15 adolescents and 5 key informants were included as showing Fig. 1. Data were collected through semi-structured in-depth interviews (IDIs) and key informant interviews (KIIs). Interview guides were developed iteratively, informed by preliminary literature reviews and pilot-tested with two adolescents and one teacher (excluded from the main study). The guides focused on five themes: peer influence, academic stress, family dynamics, Consequences, and community risk factors. Interviews were conducted in Somali by bilingual researchers trained in qualitative methods, lasting 45–75 min. Sessions were audio-recorded with participant consent, transcribed verbatim, and translated into English by certified translators. Back-translation checks ensured linguistic accuracy. Interviews were conducted by a trained male PhD candidate as well as a researcher with a background of qualitative methods. All researchers received formal instruction in ethical interviewing, transcription, and coding.

### Data analysis

NVivo 12 [13] (QSR International) was used to code and analyze all transcripts using Braun and Clarke’s six-phase thematic analysis framework. Researchers first familiarized themselves with transcripts through repeated readings. Initial coding generated 112 open codes (e.g., “pharmacy accessibility,” “exam anxiety”), which were grouped into categories such as “academic pressure”, “peer normalization.”, “Consequences”, “Motivations”, and “Family Dynamics”.

These categories were refined into five overarching themes through iterative discussion. To ensure reliability, 20% of transcripts were double-coded by an independent researcher, achieving a Cohen’s kappa coefficient of 0.85. Final themes were mapped against study objectives to ensure coherence.

### Data saturation

Data saturation was achieved after the 12th adolescent interview, as no new themes emerged in subsequent transcripts. The remaining three interviews confirmed existing patterns without introducing novel insights, indicating that the sample size was adequate for capturing the breadth of participants’ experiences.

### Trustworthiness

Data triangulation was ensured by including different participant types—adolescents, parents, teachers, and a community advocate—providing multiple perspectives on the same phenomenon. Credibility was ensured through member checking, where 10 participants validated summary interpretations of their interviews. Peer debriefing sessions with two external qualitative researchers minimized interpretive bias. Transferability was achieved by documenting thick descriptions of participant demographics, recruitment settings, and sociocultural contexts. Reflexivity was maintained through researcher journals that recorded personal biases and assumptions, particularly regarding clan dynamics and cultural stigma.

## Results

### Participant characteristics

A total of 20 participants were included in the study: 15 adolescents (aged 14–19) and 5 key informants (2 parents, 2 teachers, and 1 community leader). The majority of adolescents were male (67%, *n* = 10), reflecting broader trends in substance use demographics in Mogadishu. Most participants were enrolled in secondary education (80%, *n* = 12), while three had dropped out due to academic or socioeconomic challenges (Table 1). 

**Table 1:** Socio-Demographic characteristics of adolescent participants

Variable	Category	Frequency	Percentage
Sex	Male	10	67%
	Female	5	33%
Education Level	Secondary school	12	80%
	School dropout	3	20%
Employment	Unemployed	13	87%
	Part-time work	2	13%
Characteristic	Category	Frequency (n)	Percentage (%)
Sex	Male	10	66.7
	Female	5	33.3
Age Range (Years)	14–16	6	40.0
	17–19	9	60.0
Education Status	In school	12	80.0
	School dropout	3	20.0
Employment	Unemployed	13	86.7
	Part-time work	2	13.3

Role	Gender	Affiliation/Location
Teacher 1	Male	Secondary School – Hodan
Teacher 2	Female	Secondary School – Hamar-Weyne
Parent 1	Male	Wadajir District
Parent 2	Female	Howlwadag District
Community Advocate	Male	Hamar-Jajab District

### Themes and findings

Analysis revealed five overarching themes: Motivations and Risk Factors for Drug Misuse, Peer Influence, Academic Pressure, Family Dynamics, and Consequences of Adolescent Drug Misuse. These themes, along with their subthemes and representative quotes, are presented in Table 2.

**Table 2:** Themes, subthemes, and representative quotes

Theme	Subtheme	Illustrative Quote
Motivations and Risk Factors for Drug Misuse	Academic stress	*“Pills helped me focus.”* (17 M)
Peer Influence	Social media normalization	*“You feel left out if you don’t try them.”* (16 M)
Academic Pressure	Fear of failure	*“My family will disown me.”* (15 M)
Family Dynamics	Parental neglect	*“They’re too busy surviving.”* (14 F)
Consequences	Health risks	*“I had seizures.”* (17 M)

#### Motivations and risk factors for drug misuse

##### Academic stress and psychological burden

Adolescents frequently misused stimulants (e.g., methylphenidate) to cope with exam anxiety and academic stress. Many believed these substances enhanced focus and prolonged study hours:

*“Exams made me anxious. Pills helped me focus. I started with one tablet*,* but now I need three to study.”* — 17-year-old male student.

##### Ease of access to prescription drugs

80% of participants reported that they could purchase opioids or sedatives without a prescription from local pharmacies:

*“You can buy codeine at any pharmacy. No one asks for a prescription—just money.” *— 19-year-old female participant.

#### Peer influence and social networks

##### Social media normalization of drug use

Participants highlighted the role of social media platforms, particularly TikTok and WhatsApp, in spreading misinformation about “study drugs”:

*“Everyone posts about pills on TikTok. They call them ‘brain boosters.’ You feel left out if you don’t try them.”*— 16-year-old male student.

##### Clan-based social influence

Clan-based networks reinforced substance use, making refusal difficult:

*“Youth in the same clan often share drugs. Refusing means risking exclusion from the group.”*— Community leader.

However, a minority of participants resisted peer influence, emphasizing personal discipline:

*“My friends offered me pills*,* but I refused. I knew it was risky*,* and I didn’t want to ruin my future.”*— 18-year-old female student.

#### Academic stress and performance pressure

##### Fear of academic failure

The pressure to excel in school, compounded by limited university slots, led some adolescents to substance misuse:

*“If I fail*,* my family will disown me. Pills keep me awake to study all night.”*— 15-year-old male student.

##### Teacher observations on drug misuse in schools

Teachers noted that stimulant trading among students was common:

*“Students openly trade stimulants before exams. They see it as a shortcut to success.”*— Secondary school teacher.

#### Family and community risk factors

##### Parental neglect and economic struggles

Many adolescents reported a lack of parental supervision due to economic hardships:

*“My parents work all day. They don’t know I use pills—they’re too busy surviving.”*— 14-year-old female participant.

##### Community stigma and limited mental health support

Cultural stigma surrounding drug use and mental health deterred families from seeking help:

*“We hide drug use to avoid shame. No one wants their child labeled ‘crazy.’"*.— Parent participant.

Despite these challenges, some families actively discouraged drug use by fostering open conversations:

*“I talk to my son about drugs. He knows the risks and avoids them.”*— Parent participant.

#### Consequences of drug misuse

##### Health risks and addiction

Participants described severe health complications, including seizures and addiction:

*“I had seizures after mixing tramadol and energy drinks. I couldn’t stop—I needed more pills to function.”*— 17-year-old male student.

##### Declining academic performance

Drug misuse ultimately led to academic struggles, contrary to students’ initial expectations:

*“My grades dropped once I got addicted. Now I skip school to buy drugs.”*— 16-year-old female participant.

However, a few participants managed to quit drug use and recover academically:

*“I realized I was failing. I stopped using*,* and now I’m improving in class.”*— 17-year-old male student.

## Discussion

The findings of this study reveal that prescription drug misuse among Somali adolescents is driven by intersecting psychological, social, and structural factors. Adolescents in Mogadishu primarily misuse stimulants, opioids, and sedatives to cope with academic stress, peer pressure, and untreated mental health challenges. The ease of accessing prescription drugs without medical oversight—reported by 80% of participants—highlights systemic gaps in pharmaceutical regulation, consistent with findings from Mogadishu-based studies where pharmacies routinely dispense controlled substances illegally [14]. Peer influence, amplified by social media and clan-based networks, further normalizes drug use, mirroring trends observed in Ethiopian youth who similarly rely on peer networks for substance access [3]. However, Somalia’s post-conflict context intensifies vulnerabilities, as economic instability and parental neglect leave adolescents unsupervised, a pattern less prevalent in more stable African nations like Kenya [8].

These findings align with Bronfenbrenner’s Ecological Systems Theory. The microsystem covers adolescents’ direct interactions with family, peers, teachers, and others in everyday settings. The mesosystem reflects links between these settings, such as family–school relationships. The exosystem includes external factors that indirectly affect adolescents, like parental work conditions or local policies. The macrosystem represents cultural norms and laws, while the chronosystem captures changes over time.

The role of peer influence in drug initiation aligns with studies in Ethiopia, where clan affiliations similarly reinforce group norms [2]. However, Somalia’s context differs in the prominence of prescription drugs over traditional substances like khat, likely due to their perceived safety and availability [5]. Academic stress as a driver mirrors findings in Kenya, but Somalia’s under-resourced education system—with only 32% secondary enrollment—intensifies competition, pushing students toward extreme coping mechanisms [5].

The interplay of family neglect and socioeconomic strain reflects broader trends in fragile states. A 2023 World Bank report highlighted that 45% of Somali households rely on daily wage labor, limiting parental capacity for supervision [4]. This contrasts with middle-income African countries, where family interventions are more feasible [12].

Based on our findings, interventions should prioritize improving pharmacy regulation, integrating school-based mental health education, and promoting family engagement through community programs. Broader policy measures may be explored in future research but are beyond the direct scope of this study.

### Limitations

This study has some limitations. Its focus on Mogadishu may not reflect patterns in rural areas. Self-reported data may introduce bias due to stigma, and causal relationships cannot be determined. Future research should include longitudinal studies and rural settings for a broader perspective.

## Conclusion

This study highlights the multifaceted drivers of prescription drug misuse among Somali adolescents, including peer pressure, academic stress, family neglect, and poor pharmaceutical regulation. These findings, framed within Bronfenbrenner’s Ecological Systems Theory, underscore the complex interplay between individual, social, institutional, and cultural factors. The study contributes to the limited body of knowledge on youth substance misuse in fragile-state contexts and calls for multi-level interventions. Specifically, policies should address pharmacy regulation, school-based counseling, parental education, and culturally sensitive anti-stigma campaigns. These insights offer a foundation for future research and targeted public health strategies aimed at protecting vulnerable adolescents in Somalia and similar low-resource settings.

## Data Availability

All data generated or analyzed during this study are included in this published article [and its supplementary information files.

## Appendix

### Interview guides

**In-Depth Interview (IDI) Guide for Adolescents**.

**Sect. 1: Background and Context**.

- Can you tell me a little about yourself (age, school status, family)?
- How is your typical day like?

**Sect. 2: Drug Use History and Motivations**.

- When did you first start using prescription drugs?
- What type of drugs do you use, and how do you get them?
- What made you try these drugs for the first time?
- Do you believe these drugs help you? In what way?

**Sect. 3: Peer and Social Influence**.

- Have your friends or classmates used these drugs?
- How do social media or online platforms affect your decision?
- Do you feel pressured to use drugs to fit in?

**Sect. 4: Academic Pressure**.

- How do school expectations affect you?
- Do you think prescription drugs help you study or deal with exams?

**Sect. 5: Family and Community**.

- What kind of support do you get from your family?
- How does your family react if they know or suspect drug use?
- What is the community’s view about drug use?

**Sect. 6: Effects and Future Plans**.

- Have you experienced any side effects from using the drugs?
- Have you ever thought about quitting or sought help?

**Key Informant Interview (KII) Guide for Parents**,** Teachers**,** Community Leaders**.

**Sect. 1: Observation of Adolescent Behavior**.

- What changes have you observed in adolescent behavior in recent years?
- Have you noticed any increase in drug misuse among youth?

**Sect. 2: Risk Factors**.

- What factors do you believe contribute to prescription drug misuse?
- How do you see the role of school, family, or peer influence?

**Sect. 3: Access and Regulation**.

- How easy is it for adolescents to obtain prescription drugs in this community?

**Sect. 4: Interventions and Solutions**.

- What interventions are currently in place (if any)?
- What do you think should be done to prevent or reduce misuse?

## References

[1] United Nations Office on Drugs and Crime (UNODC). World Drug Report 2023 [Internet]. 2023. Available from: https://www.unodc.org/unodc/en/data-and-analysis/world-drug-report-2023.html

[2] Leather A, Ismail EA, Ali R, Abdi YA, Abby MH, Gulaid SA, et al. Working together to rebuild health care in post-conflict Somaliland. Lancet. 2006;368(9541):1119–25. 10.1016/S0140-6736(06)69047-8. 16997666 10.1016/S0140-6736(06)69047-8

[3] Asmelashe Gelayee D, Binega G. Assessment of medication use among university students in Ethiopia. TheScientificWorldJournal. 2017;2017:4530183. 10.1155/2017/4530183. 28393101 10.1155/2017/4530183 PMC5368397

[4] Somali Ministry of Education. National education statistics report. Mogadishu: Federal Government of Somalia; 2023.

[5] Ibrahim M, Rizwan H, Afzal M, et al. Mental health crisis in somalia: a review and a way forward. Int J Ment Health Syst. 2022;16(1):12. 10.1186/s13033-022-00525-y. 35139873 10.1186/s13033-022-00525-y PMC8827242

[6] Al-Juhaishi T, Al-Kindi S, Gehani A, Khat. A widely used drug of abuse in the Horn of Africa and the Arabian peninsula: review of literature. Qatar Med J. 2013;2012(2):1–6. PMID: 25003033; PMCID: PMC3991038. 25003033 10.5339/qmj.2012.2.5 PMC3991038

[7] World Bank. Somalia Economic Update: Building Resilience in a Fragile Context [Internet]. 2023. Available from: https://www.worldbank.org/en/country/somalia/overview

[8] Atwoli L, Mungla PA, Ndung’u MN, et al. Prevalence of substance use among college students in eldoret, Western Kenya. BMC Psychiatry. 2011;11(1):34. 10.1186/1471-244X-11-34. 21356035 10.1186/1471-244X-11-34 PMC3053226

[9] Bwanika JM, Hawkins C, Kamulegeya L, Afr, et al. J Psychiat. 2022;28(0):a1690. 10.4102/sajpsychiatry.v28i0.1690. 10.4102/sajpsychiatry.v28i0.1690 PMC9210188 35747338

[10] Sequeira M, Singh S, Fernandes L, Gaikwad L, Gupta D, Chibanda D, et al. Adolescent health series: the status of adolescent mental health research, practice and policy in sub-Saharan africa: A narrative review. Trop Med Int Health. 2022;27(9):758–66. 10.1111/tmi.13802. 35906997 10.1111/tmi.13802 PMC9544168

[11] Bronfenbrenner U. The ecology of human development: experiments by nature and design. Cambridge, MA: Harvard University Press; 1979.

[12] National Authority for the Campaign Against Alcohol and Drug Abuse (NACADA). National Guidelines on Drug Use Prevention [Internet]. 2021. Available from: [Internet].

[13] Dhakal K, NVivo. J Med Libr Assoc. 2022;110(2):270–2. 10.5195/jmla.2022.1271. PMID: 35440911; PMCID: PMC9014916. 35440911 10.5195/jmla.2022.1271 PMC9014916

[14] Save the Children. Health Camp: An Alternate Model to Deliver Health and Nutrition Services Ensuring Equity and Accessibility for Areas Controlled by Non-State Armed Actors in Somalia [Internet]. Available from: https://resourcecentre.savethechildren.net/document/health-camp-an-alternate-model-to-deliver-health-and-nutrition-services-ensuring-equity-and-accessibility-for-areas-controlled-by-non-state-armed-actors-in-somalia/

